# Nasal negative pressure oscillatory therapy versus oscillatory positive expiratory pressure for airway clearance in patients with acute exacerbations of bronchiectasis (NNPO-BE): a multicentre, randomised, crossover non-inferiority trial protocol

**DOI:** 10.3389/fmed.2026.1788242

**Published:** 2026-02-24

**Authors:** Siyuan Wang, Zhaobin Zhou, Xiaobin Ma, Yang Yang, Jieping Lei, Qumu Shiwei, Beiyao Gao, Peijian Wang, Man Le, Shan Jiang, Ting Yang

**Affiliations:** 1 Department of Rehabilitation Medicine, China-Japan Friendship Hospital, Beijing, China; 2 Department of Pulmonary and Critical Care Medicine, The First Affiliated Hospital of Nanchang University, Nanchang, Jiangxi, China; 3 Department of Pulmonary and Critical Care Medicine, Shandong Provincial Hospital Affiliated to Shandong First Medical University, Jinan, Shandong, China; 4 Department of Rehabilitation Medicine, The First People's Hospital of Yunnan Provincial, Kunming, Yunnan, China; 5 Department of Clinical Research and Data Management, Center of Respiratory Medicine, China-Japan Friendship Hospital, Beijing, China; 6 National Center for Respiratory Medicine, Beijing, China; 7 National Clinical Research Center for Respiratory Diseases, Beijing, China; 8 Institute of Respiratory Medicine, Chinese Academy of Medical Sciences, Beijing, China; 9 Department of Pulmonary and Critical Care Medicine, Center of Respiratory Medicine, China-Japan Friendship Hospital, Beijing, China; 10 Department of Clinical Pharmacology Research Center, Peking Union Medical College Hospital, Beijing, China

**Keywords:** acute exacerbation of bronchiectasis, airway clearance techniques, multicenter, nasal negative pressure oscillatory therapy, non-inferiority, randomized crossover trial

## Abstract

**Background:**

Airway clearance techniques (ACTs) are a cornerstone of bronchiectasis management, particularly during acute exacerbations characterized by mucus retention and impaired mucociliary clearance. However, conventional ACTs are frequently limited by patient tolerance, reliance on skilled personnel and variable adherence in real-world settings. Nasal negative pressure oscillatory airway clearance represents a novel, non-invasive strategy designed to enhance expiratory flow bias and promote mobilization of airway secretions.

**Objectives:**

To assess whether nasal negative pressure oscillatory (NNPO) airway clearance is non-inferior to standard airway clearance therapy, with oscillatory positive expiratory pressure (OPEP) used as the conventional comparator, in hospitalized patients with acute exacerbations of bronchiectasis.

**Methods:**

This is a prospective, multicenter, randomized crossover trial conducted across four tertiary hospitals in China. Eligible participants will receive NNPO and OPEP therapy in random order over four consecutive days. The primary outcome is sputum wet weight collected during and within 30 min after each treatment session. Secondary outcomes include lung function, peripheral oxygen saturation, patient-reported efficacy and comfort, and adverse events.

**Conclusion:**

This trial will evaluate the short-term efficacy and safety of nasal negative pressure oscillatory airway clearance compared with conventional airway clearance therapy in hospitalized patients with acute exacerbations of bronchiectasis, providing evidence to inform clinical decision-making in acute management.

**Systematic review registration:**

ChiCTR2500105694 PID: 266607, https://www.chictr.org.cn/bin/project/edit?pid=266607.

## Introduction

Bronchiectasis is a chronic respiratory disorder characterized by irreversible bronchial dilatation, persistent airway inflammation and recurrent infection, frequently accompanied by excessive sputum production and impaired mucociliary clearance. Acute exacerbations are commonly driven by mucus retention and airway obstruction, resulting in worsening respiratory symptoms, increased infection burden and a high risk of hospitalization (1–3), Effective airway clearance is therefore a central component of management during acute exacerbations of bronchiectasis (4, 5).

The European Respiratory Society (ERS) guidelines for bronchiectasis recommend that all patients with bronchiectasis receive regular airway clearance therapy (ACT), including those who expectorate sputum regularly as well as those who have difficulty expectorating sputum or do not expectorate sputum daily (6). Nevertheless, the effectiveness of current airway clearance therapies (ACTs)—including ACBT, autogenic drainage, postural drainage, PEP/OPEP devices, and manual chest physiotherapy—is often influenced by patient preferences, the pathological heterogeneity of bronchiectasis, as well as patient fatigue, discomfort, dependence on trained physiotherapists, and inconsistent adherence (7, 8), particularly in hospitalized patients with acute illness. Recent European Respiratory Society statements emphasize the need for individualized, standardized and evidence-based airway clearance strategies to address the marked heterogeneity of bronchiectasis (9).

Oscillatory positive expiratory pressure (OPEP) therapy is a widely used conventional airway clearance technique that requires patients to exhale actively against resistance. The expiratory resistance generates positive airway pressure, which helps prevent premature small airway collapse during forced exhalation. In addition, oscillatory airflow produced by an internal vibrating valve reduces mucus viscoelasticity, disrupts mucus–airway wall adhesion, and enhances expiratory flow bias, thereby facilitating the transport of airway secretions toward the central airways (10).

Nasal negative pressure oscillatory therapy (NNPO), developed in China, represents a novel airway clearance approach that differs from OPEP in several aspects, including pressure characteristics and patient involvement. NNPO delivers controlled negative pressure combined with oscillatory airflow via the nasal route, integrating two airway clearance mechanisms: oscillatory airflow, which disrupts mucus–airway wall adhesion and improves mucus rheology, and expiratory negative pressure, which increases peak expiratory flow and augments expiratory flow bias, thereby promoting the transport of secretions from peripheral to central airways (11, 12). Importantly, NNPO generates continuous effects throughout the respiratory cycle and is largely independent of active patient effort, potentially offering more consistent airway clearance during acute illness.

Similar intermittent negative pressure–based bronchial drainage devices, such as Simeox®, have demonstrated improvements in sputum properties and diaphragmatic excursion in patients with cystic fibrosis, supporting the physiological rationale of this approach (13). In addition, intermittent negative pressure oscillatory airway clearance combined with conventional chest physiotherapy has been shown to improve lung function during acute pulmonary exacerbations in pediatric cystic fibrosis populations, with good safety and tolerability (14).

Despite these encouraging findings, high-quality clinical evidence for NNPO during acute exacerbations of bronchiectasis remains limited, and its comparative effectiveness against OPEP therapy in hospitalized patients has not been systematically evaluated. Therefore, this study aims to assess the short-term efficacy and safety of a novel NNPO bronchial drainage device (Lung Clear Device; ZD-HY) compared with OPEP therapy in hospitalized patients with acute exacerbations of bronchiectasis.

## Methods

### Trial objectives

The primary objective of this trial is to evaluate the short-term efficacy of nasal negative pressure oscillatory airway clearance delivered via a novel bronchial drainage device, compared with OPEP, in hospitalized patients experiencing acute exacerbations of bronchiectasis. Efficacy will be assessed by comparing sputum clearance and other airway clearance–related outcomes between interventions.

Secondary objectives are to assess the safety, tolerability and feasibility of nasal negative pressure oscillatory airway clearance in the acute hospital setting, as well as patient acceptance of the intervention. In addition, the study aims to explore the effects of NNPO on pulmonary function during hospitalization.

### Trial design

This prospective, multicenter, randomized, crossover, non-inferiority trial aims to evaluate the efficacy of a novel airway clearance technique, nasal negative pressure oscillatory (NNPO) therapy, in hospitalized patients. The protocol is developed and reported in accordance with the SPIRIT 2025 guidelines (15). To minimize inter-individual variability in sputum production and airway clearance capacity, a self-controlled crossover design is employed, with each participant receiving both interventions and serving as their own control. NNPO therapy is compared with conventional airway clearance using oscillatory positive expiratory pressure (OPEP).

**Figure 1:**
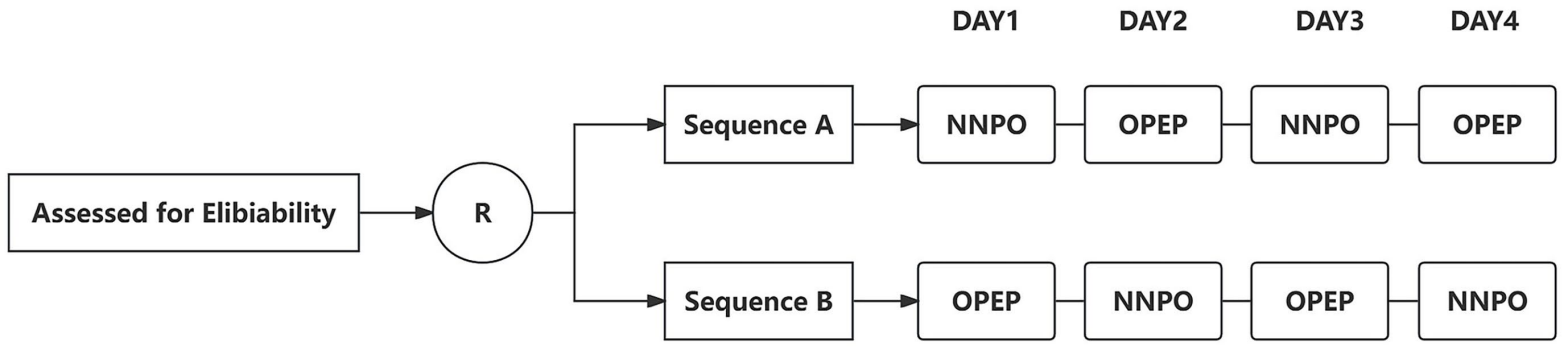
Study design and intervention sequence of the randomized crossover trial.

The study is conducted over a continuous 4-day intervention period. Participants receive one intervention on Days 1 and 3 and the alternate intervention on Days 2 and 4. Two treatment sequences are predefined: Sequence A, in which NNPO is administered on Day 1 followed by OPEP on Day 2, and Sequence B, in which OPEP is administered on Day 1 followed by NNPO on Day 2. The same intervention sequence is repeated on Days 3 and 4 to enhance the reliability of treatment comparisons. Allocation to Sequence A or Sequence B is performed using a computer-generated randomization schedule (Figure 1).

The primary objective of the trial is to determine whether NNPO therapy, as a short-term intervention, is non-inferior to conventional OPEP therapy with respect to sputum clearance.

### Randomization and blinding

Randomization was performed using a computer-generated random sequence, which assigned participants to one of two intervention sequences (NNPO first or OPEP first) in a 1:1 ratio. Allocation was concealed using sealed, opaque envelopes prepared by an independent researcher not involved in participant recruitment, intervention delivery, or outcome assessment. Due to the nature of the interventions, participants and treating physiotherapists were not blinded. However, outcome assessors and data analysts were blinded to treatment allocation throughout the study.

### Washout considerations

A formal washout period will not be incorporated in this study because hospitalized patients with acute exacerbation of bronchiectasis may experience rapid changes in airway secretion burden and clinical status over a short period. In addition, suspending airway clearance during a washout period would be unethical, as secretion clearance is necessary in this patient population, making a prolonged washout period impractical. However, to minimize potential carryover effects, each intervention will be performed on a separate day.

### Trial setting

The trial will be conducted across four tertiary teaching hospitals in China. The China–Japan Friendship Hospital in Beijing will serve as the coordinating center, with participating sites including the First Affiliated Hospital of Nanchang University (Nanchang), Shandong Provincial Hospital Affiliated to Shandong First Medical University (Jinan), and The First People’s Hospital of Yunnan Provincial (Kunming). All participating centers have established respiratory rehabilitation teams and experience in airway clearance interventions. Prior to study initiation, investigators at all sites will receive unified training on the study protocol to ensure consistency and standardization of trial procedures.

### Eligibility and withdrawal criteria

Participants eligible for inclusion are adults aged 18–75 years with radiologically confirmed bronchiectasis on high-resolution computed tomography, hospitalized for acute exacerbation (defined as worsening of ≥3 key symptoms for ≥48 h requiring treatment change), and able to provide written informed consent. Participants are excluded if they have severe cardiovascular disease (e.g., decompensated heart failure, unstable arrhythmia), recent massive hemoptysis (>100 mL in a single episode or >300 mL within 24 h), severe tympanic membrane pathology or recent otologic surgery, or cognitive/psychiatric conditions impairing understanding or cooperation.

Allocated interventions may be withdrawal under the following circumstances: serious adverse events or clinically significant deterioration related to the intervention; development of contraindicating conditions (e.g., hemoptysis, acute cardiorespiratory instability, severe intolerance); participant request for withdrawal; or inability to safely continue as judged by the investigator. In such cases, participants will continue to receive standard medical care according to routine practice (Table 1).

**Table 1:** Eligibility and withdrawal criteria for study participants.

Category	Criteria
Inclusion criteria	Age 18–75 years Radiologically confirmed bronchiectasis on high-resolution CT Hospitalization for acute exacerbation (≥3 symptom worsening for ≥48 h requiring treatment change) Ability to provide written informed consent
Exclusion criteria	Severe cardiovascular disease (decompensated heart failure, unstable arrhythmia) Recent massive hemoptysis (>100 mL single episode or >300 mL/24 h) Severe tympanic membrane pathology or recent otologic surgery Cognitive or psychiatric impairment affecting understanding or cooperation
Withdrawal criteria	Serious adverse events or clinically significant deterioration related to intervention (including but not limited to new-onset hemoptysis, treatment-related pneumothorax, or new tympanic membrane injury, hearing impairment or severe intolerance.) Participant request to withdraw from intervention or study Investigator judgement that continuation is unsafe (for example, uncontrolled hypertension, worsening heart failure, significant arrhythmia, sustained oxygen desaturation, or acute infectious exacerbation such as high fever)

## Intervention

Participants received both interventions according to a randomized crossover sequence, with each supervised session performed once daily in the morning.

### Nasal negative pressure oscillatory (NNPO) therapy

NNPO therapy was delivered using a nasal device that generated oscillatory negative pressure airflow in enhanced mode (flow rate: 2600 mL·min^−1^ ± 500 mL·min^−1^; pressure range: 320–900 Pa; sinusoidal waveform at 50 Hz). During NNPO therapy, participants wore a nasal interface and performed repeated standardized breathing maneuvers consisting of a slow deep inspiration, a 2–3-s end-inspiratory breath hold, followed by a slow expiration while receiving oscillatory negative pressure airflow via the nasal route.

Three consecutive breathing maneuvers were performed, followed by 30–40 s of quiet breathing; this sequence constituted one cycle and lasted approximately 3 min.

### Oscillatory positive expiratory pressure (OPEP) therapy

OPEP therapy was administered using an oscillatory positive expiratory pressure device (PE100, Respiratory Rehabilitation Device, e-Linkcare Meditech Co., Ltd), with expiratory pressure set between 10 and 20 cm H₂O in accordance with manufacturer recommendations. Participants followed the same standardized breathing structure as in the NNPO intervention, including a slow deep inspiration, a 2–3-s end-inspiratory breath hold, and expiration performed through the OPEP device.

The cycle structure, timing, and session organization were identical to those used during NNPO therapy; the only difference between interventions was the expiratory phase, during which expiration was performed through the OPEP device rather than during nasal negative pressure delivery.

### Session structure and airway clearance maneuvers

**Figure 2:**
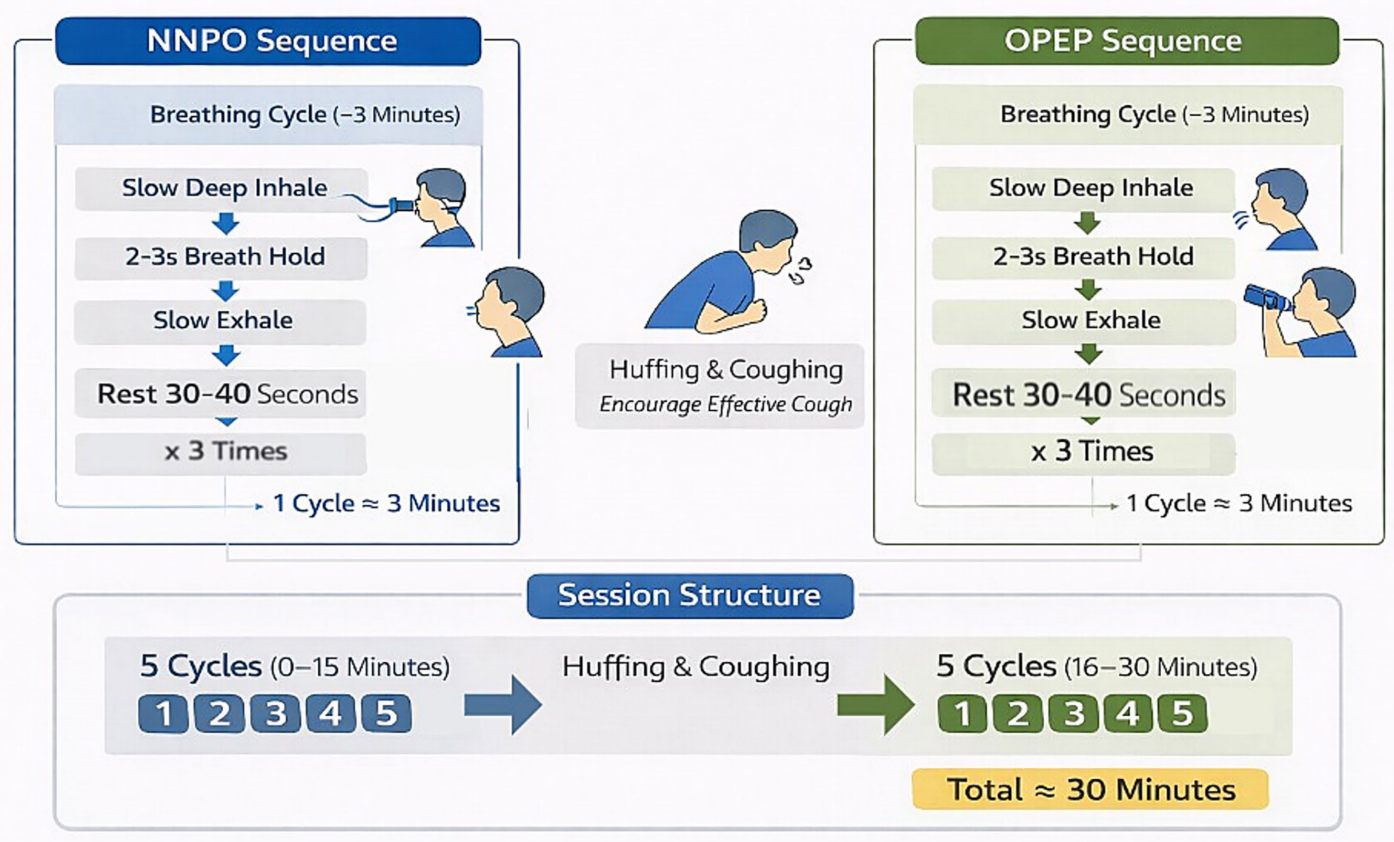
Structure of the supervised NNPO and OPEP therapy session.

Each supervised session consisted of two sets of five cycles. After completion of the first five cycles (approximately 15 min), participants were encouraged to perform huffing and coughing to facilitate airway secretion clearance, followed by a short rest before commencing the second set. The total session duration was approximately 30 min. Huffing was systematically instructed at 15 min after session initiation and again at the end of the session, with additional coughing encouraged as needed (Figure 2).

Device settings were maintained throughout each session and adjusted only to ensure patient comfort and tolerance.

In addition to the supervised intervention sessions, all participants received instruction in general airway clearance techniques without the use of devices as part of routine care. These techniques referred to commonly used non-device strategies, such as appropriate mobilization, breathing control, huffing, and coughing. Participants were not restricted in the frequency or type of self-performed airway clearance techniques outside the supervised intervention sessions.

### Outcomes and assessment schedule

The primary outcome was sputum wet weight, collected during each treatment session and for up to 30 min after session completion. Sputum was collected into pre-weighed, sealed sterile containers and immediately weighed using a calibrated electronic scale. Collection was performed using a standardized procedure under the supervision of study personnel throughout the airway clearance process. During collection, participants were instructed to avoid swallowing sputum when present and to expectorate it promptly for collection. Study personnel made real-time judgments based on the pattern of expectoration and avoided collecting secretions following throat clearing or intentional saliva generation. For participants with viscous oral secretions that might increase the risk of saliva contamination, mouth rinsing with water was permitted prior to treatment to reduce oral contamination.

Secondary outcomes include: (1) Lung function—FEV₁, FVC, FEV₁/FVC, MEF₇₅, MEF₅₀, MEF₂₅, and MMEF₇₅/₂₅—measured before and 30 min after each daily treatment session. Lung function parameters were assessed using a respiratory rehabilitation device (PE100, e-Linkcare Meditech Co., Ltd.); (2) peripheral oxygen saturation (SpO₂/FiO₂) measured at rest before treatment, during the final minute of each session, and 30 min post-treatment; (3) patient-reported treatment efficacy and comfort assessed using 10-cm visual analogue scales after completion of all treatment days; and (4) the incidence of adverse events during or immediately after each session (Table 2).

**Table 2:** Assessment schedule for primary and secondary outcomes.

Outcome	Time points of assessment
Sputum wet weight (g)	Collected during each treatment session and up to 30 min post-intervention
Lung function (FEV1, FVC, FEV1/FVC, MEF75, MEF50, MEF25, MMEF75/25)	Measured immediately before each daily treatment session and 30 min after completion
Peripheral oxygen saturation (SpO₂/FiO₂)	Recorded at baseline (pre-treatment), during the final minute of the session, and 30 min post-treatment
Patient-reported treatment efficacy (VAS 0–10)	Assessed after completion of all daily treatment sessions
Patient-reported comfort (VAS 0–10)	Assessed after completion of all daily treatment sessions
Adverse events	Monitored during and immediately following each treatment session

### Sample size calculation

The sample size was calculated based on the primary outcome, sputum wet weight, using a non-inferiority design. A non-inferiority margin of 1.5 g was prespecified, as justified above. Variability estimates were obtained from a preliminary pilot study, which indicated a within-subject standard deviation of approximately *σ* g for wet sputum weight following airway clearance interventions.

Assuming a true mean difference of 0 g between NNPO and OPEP, a one-sided significance level (*α*) of 0.025, and 80% power, a minimum of 38 participants was required to demonstrate non-inferiority in this crossover study. To allow for potential dropouts or incomplete data, the target sample size was increased to 42 participants.

## Statistical analysis

All statistical analyses will be performed using R software (version 4.3; R Foundation for Statistical Computing, Vienna, Austria). Normality of continuous variables will be assessed using the Shapiro–Wilk test. Normally distributed variables will be presented as mean ± standard deviation (SD), while non-normally distributed variables will be reported as median (interquartile range, IQR). Categorical variables will be summarized as counts and percentages.

Between-treatment differences in wet sputum weight will be analyzed using linear mixed-effects models (LMMs), with treatment group as a fixed effect and participant as a random effect. In the absence of a statistically significant difference, a non-inferiority analysis will be performed comparing NNPO therapy with OPEP therapy. The non-inferiority margin is set at 1.5 g. As no established margin exists for short-term wet sputum weight, this threshold was determined *a priori* based on evidence suggesting a clinically meaningful 24-h sputum weight change of approximately 6.4 g following airway clearance interventions (16), combined with pilot data demonstrating minimal between-treatment differences. Non-inferiority will be concluded if the lower bound of the 95% confidence interval (CI) for the NNPO–OPEP difference exceeds −1.5 g.

Lung function parameters and oxygenation (SpO₂/FiO₂) will also be analyzed using LMMs to assess pre–post intervention changes and treatment-by-time interactions. Change scores (*Δ*) will be calculated and compared between interventions.

Patient-reported outcomes, including perceived efficacy and comfort, will be compared using independent-samples t-tests or Mann–Whitney U tests, as appropriate. All statistical tests will be two-sided unless otherwise specified. A *p* value < 0.05 will be considered statistically significant, except for the non-inferiority analysis of wet sputum weight, which will use a one-sided significance level of 0.025.

## Handling of missing data

Given the crossover design and short intervention period, mixed-effects models were considered sufficient to handle incomplete data without formal imputation.

## Data monitoring

Given the short duration and low-risk nature of the interventions, and the absence of planned interim analyses, no independent data monitoring committee has been established. Study conduct and safety are overseen by the coordinating centre. All adverse events are reviewed promptly according to predefined data monitoring procedures, which include identification and documentation of adverse events, assessment of severity and causality, implementation of appropriate medical management when necessary, and reporting to the study team.

## Ethics and dissemination

The study protocol has been approved by the Ethics Committee of the China–Japan Friendship Hospital (approval number: 2025-KY-039) and the institutional review boards of all participating centres. The trial has been prospectively registered with the Chinese Clinical Trial Registry (ChiCTR) (registration number: ChiCTR2500105694; registered on 9 July 2025). Written informed consent will be obtained from all participants prior to enrolment. The study will be conducted in accordance with the Declaration of Helsinki and Good Clinical Practice guidelines.

Study results will be disseminated through publication in peer-reviewed scientific journals and presentation at national and international conferences. Authorship will be determined in accordance with international authorship guidelines. No restrictions on publication are imposed by the funding body.

## Technical flowchart

**Figure 3:**
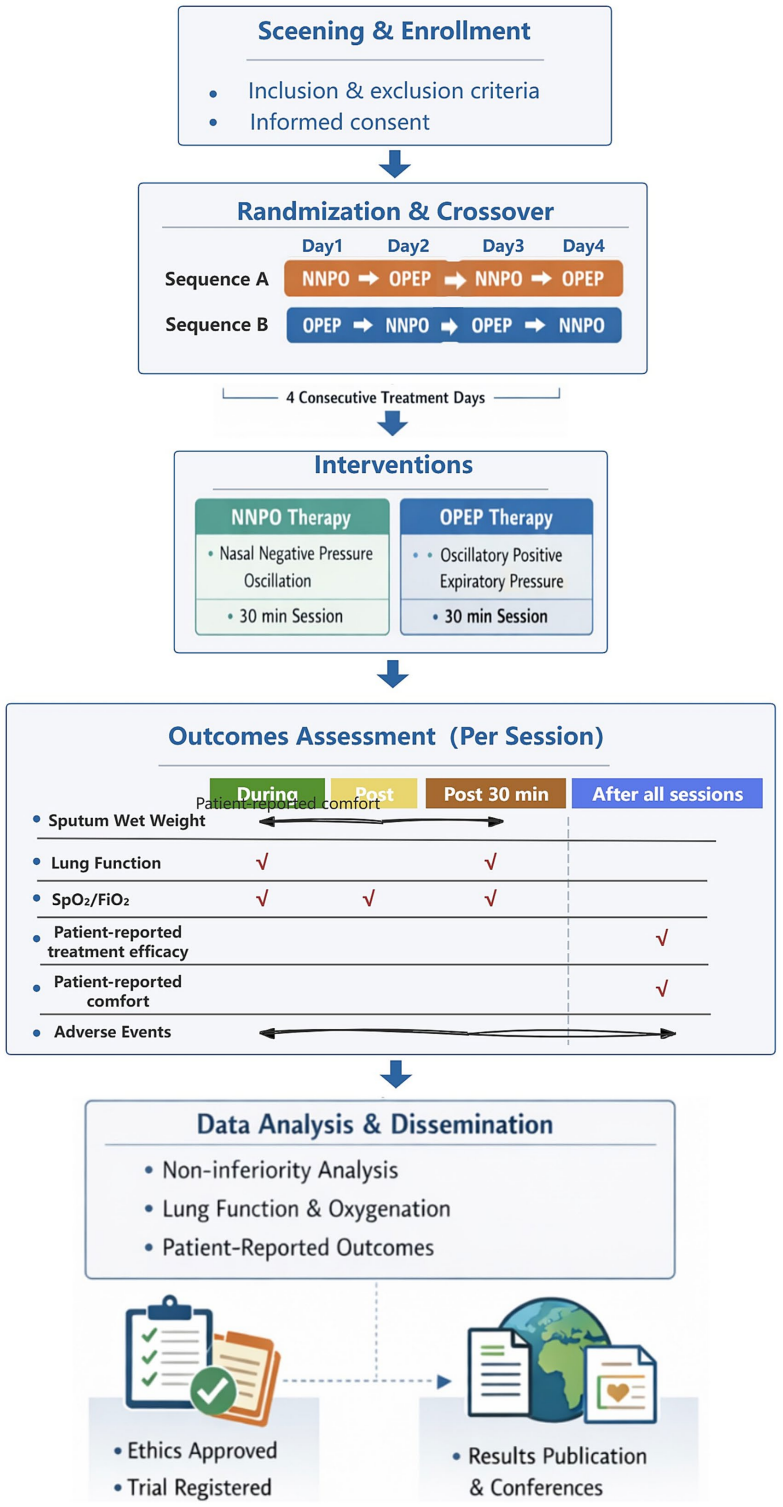
Technical flowchart of the present study.

To visually summarize the overall study design, interventions, and outcome assessments, a comprehensive technical flowchart was created (Figure 3).

## Discussion

Airway clearance is essential in managing acute exacerbations of bronchiectasis, where mucus hypersecretion and impaired clearance worsen symptoms and increase hospitalization risk (9, 17). Despite guideline recommendations, high-quality evidence comparing different airway clearance techniques in hospitalized patients is limited (15, 18). This multicenter, randomized crossover trial aims to scientifically evaluate the short-term efficacy and safety of a novel nasal negative pressure oscillatory device (NNPO) and to determine its non-inferiority relative to conventional OPEP therapy.

This study’s design features enhance its potential impact. The crossover design allows each participant to serve as their own control, reducing inter-individual variability in sputum production and airway characteristics, while the multicenter approach across four tertiary hospitals in China improves generalizability. Standardized sputum collection during and after each session provides objective measurement of immediate airway clearance, complemented by secondary outcomes including spirometry, oxygenation, and patient-reported efficacy and comfort.

NNPO acts throughout the respiratory cycle independently of patient effort, combining oscillatory airflow and negative expiratory pressure to mobilise peripheral airway secretions and enhance expiratory flow bias (19). Demonstrating non-inferiority or superiority relative to OPEP could support its use as a practical alternative or adjunct, especially for patients with fatigue, discomfort, or difficulty performing conventional techniques.

### Limitations

Limitations include the short intervention period, which precludes assessment of long-term outcomes such as exacerbation recurrence or hospital stay, and the inability to blind participants or therapists. Nonetheless, the use of objective primary outcomes and standardized protocols is expected to minimize bias. Overall, this study will provide high-quality evidence on the short-term efficacy and safety of NNPO and inform evidence-based, individualized airway clearance strategies in hospitalized patients with acute bronchiectasis exacerbations.

In addition, some anticholinergic agents such as ipratropium bromide have been suggested to reduce bronchial secretions (20), which could theoretically influence sputum volume, as well as by the natural clinical course during treatment, in which sputum volume may gradually decrease over time. From an ethical and practical perspective, it was neither feasible nor appropriate to restrict or modify clinically indicated medications in hospitalized patients, and such variability therefore reflects real-world clinical practice. To mitigate the potential bias introduced by these unavoidable factors, this study employed a randomized crossover design, allowing each participant to serve as their own control, focused on sputum expectoration following a single treatment session, and conducted all interventions and assessments at a fixed time each morning. Nevertheless, residual confounding related to concurrent treatments cannot be completely excluded and is acknowledged as a limitation of the study.
