# Supplementary material for: Nasal negative pressure oscillatory therapy versus oscillatory positive expiratory pressure for airway clearance in patients with acute exacerbations of bronchiectasis (NNPO-BE): a multicentre, randomised, crossover non-inferiority trial protocol
Source: Front Med (Lausanne). 2026 Feb 24;13:1788242. doi: 10.3389/fmed.2026.1788242 (PMC12971644; doi:10.3389/fmed.2026.1788242)
Supplement: Supplementary file 1 [file Data_Sheet_1.pdf]

清肺仪 ZD-HY型

Lung Clear Device ZD-HY

Name of registrant/after-sales service unit: Beijing Tongxin Zhongda Medical Equipment Co., Ltd

Registrant's residence: Room B509, Building 1, No. 2A, Jinfu Road, Economic Development Zone, Daxing District, Beijing

Telephone/after-sales service telephone of registrant: 010-62563856

Name of manufacturer: Beijing Tongxin Zhongda Medical Equipment Co., Ltd

Residence of the manufacturer: Room B509, Building 1, No. 2A, Jinfu Road, Economic Development Zone, Daxing District, Beijing

Address: Room B509, Building 1, No. 2A, Jinfu Road, Economic Development Zone, Daxing District, Beijing

Telephone of manufacturer: 010-62563856

Zip code: 102600

Website: www.txzd.com

Production License: Beijing Drug Administration Supervision Production License of Medical Device No. 20200042

Registration Certificate of Medical Device: Beijing Medical Device Registration Approval 20182140104

Registration Certificate of Medical Device: Beijing Medical Device Registration Approval 20182140104

清肺仪

LUNG CLEAR DEVICE

Instruction for use of ZD–HY product

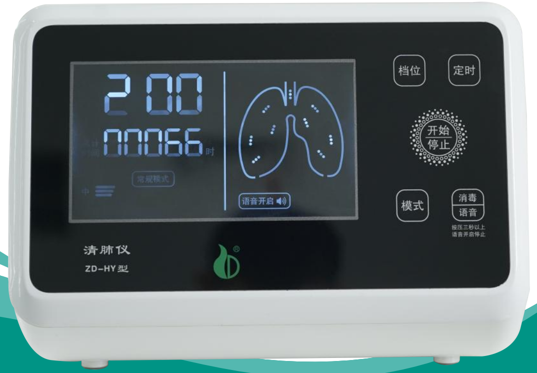

Dear Users:
Thank you for using Lung Clear Device. Please read this manual carefully for yourspeedy recovery.

Introduction

Dear Users:

Thank you for using Lung Clear Device. Please read this manual carefully for yourspeedy recovery.

Contents

I. Basic product information	02
Il. Registrant Information and After-sales Service Unit	02
Ill. Product Introduction	02
IV. Composition of product structure	03
V.Instrument performance index	04
VI. Scope of application	05
VIIContraindications	05
VIl. Introduction of product structure, use, installation and cleaning methods	05
IX.Treatment time and course	14
Ⅹ.Precautions	15
XI. Warm Tips and Maintenance Methods	16
Ⅻ.General Troubleshooting	17
XII. Safety Warning	17
XIV. Service life and maintenance of products	17
XV. Service Pledge	18
XVI. Transportation and storage conditions	18
XVII. Manufactrer's Declaration on Electromagnetic Compatibility	18
XVII. Description of Replaceable Parts	24
XIX.Symbol Description	24
XX.Packing list	25

I. Basie produet information

Product name: Lung Clear Device

Model: ZD-HY

Production date and production serial number: see label

Registration Certificate of Medical Device:Beijing Medical Device Registration Approval20182140104

II. Registrant Information and After–sales Service Unit

Registrant/manufacturer: Beijing Tongxin Zhongda Medical Equipment Co. Ltd

Address: B509, Building 1, No.2 Jinfu Road, Daxing District Economic Development Zone, Beijing

Production address: B509, Building 1, No.2 Jinfu Road, Daxing District Economic Development

Zone, Beijing. After-sales service unit: Beijing Tongxin Zhongda Medical Equipment Co. Ltd

Tel: 010-62563856. Zip code: 102600

Website: www.txzd.com

Production License No.: JYJXSCX No.20200042

III. Product Introduction

Negative pressure pulsating Lung Clear Device was developed in 1990, and Lung Clear Device ZD-HY is one of ZD series products, which mainly solves the treatment problem of respiratory dysfunction caused by difficulty in expectoration and exhaust in the treatment of obstructive respiratory diseases, and has achieved good therapeutic effects of improving ventilation and gradually restoring respiratory function. Lung Depurating Apparatus, without any drugs, has scientifically, safely and effectively achieved a major breakthrough in expectoration and exhaust in the medical field of respiratory diseases by using "negative pressure pulse therapy", and has become the first at home and abroad. In 1992, Lung Clear Device obtained the Patent Certificate of Invention of the People's Republic of China (Patent No.: ZL 92104 625.1)

Lung Clear Device uses "negative pressure pulsating oscillating airflow" to effectively solve the problem of expectoration and ventilation, eliminate respiratory disorders and improve respiratory function. The clinical prac-

tice of Lung Clear Device for more than 20 years has proved that through sputum excretion and exhaust, the medical problems of difficult dissipation of inflammation, repeated infection, increased respiratory resistance and continuous attenuation of lung function caused by sputum blocking airway can be solved. Only by solving the airway obstruction of patients can we completely diminish inflammation, improve asthma and hypoxia, and avoid repeated infection and gradual recovery of respiratory function. Lung Clear Device provides a non-invasive and drug-free rehabilitation treatment for clinical treatment. Lung Clear Device ZD-HY scientifically according to the physiological characteristics of human respiratory system, uses the continuously open "negative pressure pulsating oscillating airflow" to form negative pressure in nasal cavity. With the normal breathing of human body, the

vibration wave of 3000 times/min and the displacement of 3 liters/min are effectively transmitted to respiratory tract at all levels through respiratory airflow oscillation through nasal cavity, which promotes and improves mucociliary movement, makes sputum and residual gas discharge smoothly, achieves the purpose of clearing lung heat and regulating qi, and plays a role in dredging airway, improving hypoxia and gradually restoring lung function.

Lung Clear Device ZD-HY has two kinds of conventional mode and enhanced mode. In the conventional mode, "high, medium and low" three gears are switched, while in the enhanced mode, there is only one gear, but the reciprocating motion of pulsating airflow is increased, the amplitude is enhanced, and the expectoration effect is enhanced. The product is customized LCD display, easy to operate, voice opening and closing function, timing operation, can display accumulated time, and add disinfection box function, so as to facilitate patients to use Lung Clear Device.

IV. Composition of product structure

It consists of main engine, catheter (oxygen inhalation pipe) and gas-liquid separator.

V. Instrument performance index

Rated voltage: 220V +10%

Rated frequency: 50HZ + 2%

Output extraction flow:

In normal mode

High grade: 3000ml/min 500 ml/min;

Mid-range: 2500ml/min 500 ml/min;

Low grade: 1000ml/min 300 ml/min;

In enhanced mode: 2600ml/min 500 ml/min;

Negative pressure range: negative pressure range: 320-900Pa.

Noise: The noise of Lung Clear Device should be no more than 45dB.

Continuous working time: The default unscheduled state continuous working time is 24 hours, and the timing state is calculated according to the timing time.

Treatment mode: conventional mode and enhanced mode; In normal mode, the gear function is divided into three working states: high, medium and low, while in enhanced mode, there is only one working state.

ZD-HY ultraviolet protection function: the disinfection box is opened by magnetic attraction, and a limited safety switch is placed to avoid the leakage of ultraviolet light at the moment of opening

the door.

Performance requirements of ZD-HY ultraviolet disinfection box:

(1) UVC3535 deep ultraviolet lamp bead is adopted;

(2) The power of ultraviolet lamp is 2-4VA;

(3) Irradiation intensity is not less than 70uW/cm2;

(4) The preset irradiation time is 10min;
Other notes:
Total rated power: 24VA
Classification of electrical safety: Class II and type B
Safety requirements: electrical safety meets the requirements of GB/9706.1-2007;
Electromagnetic compatibility in accordance with YY0505-2012 Medical Electrical Equipment
Part 1-2:
General safety requirements and standard electromagnetic compatibility requirements and tests.

VI. Scope of application

This product can improve the airway obstruction caused by bronchiectasis and chronic obstructive
pulmonary disease, and help to clear the sputum in respiratory tract.

VII. Contraindications

Severe cardiopulmonary failure rescue patients, pneumothorax patients, bronchiectasis patients
with massive hemoptysis and psychotic patients are prohibited; Critically ill patients should be
used under the guidance of experts and doctors.

VIII. Introduction of product structure, use, installation and cleaning methods

Introduction of product structure:
1. Lung Clear Device ZD-HY front LCD screen and 5-key touch function button, with product name and logo icon at the bottom of the screen (see Figure 1);

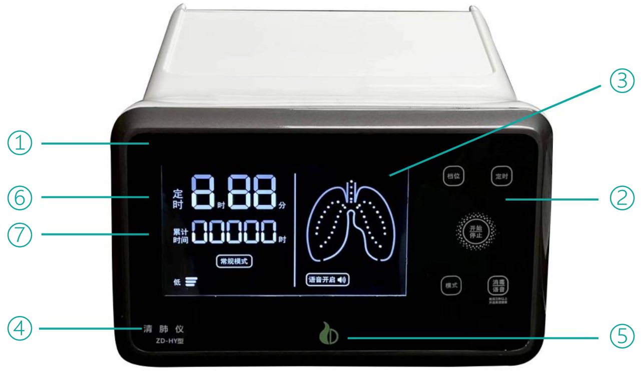

Figure 1

① Liquid crystal panel
② 5-digit function keys
③ Pulmonary angiogram
④ Product name and model
⑤ LOGO icon
⑥ Timing display
⑦ Cumulative time display

2. The back of Lung Clear Device ZD-HY is provided with a power cord and a power switch (see Figure 2);

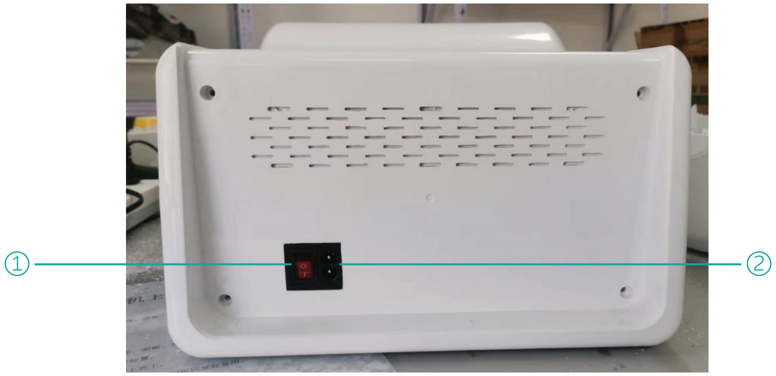

Figure 2

① Power switch
② Power cord

3. There is an ultraviolet disinfection box on the left side of Lung Clear Device ZD-HY. The disinfection box is opened by magnetic attraction, and a limited safety switch is placed to avoid the instant leakage of ultraviolet light (see Figures 3 and 4);

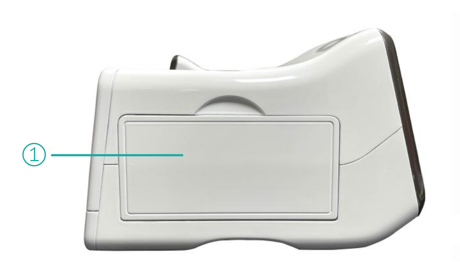

Figure 3
① Disinfection box

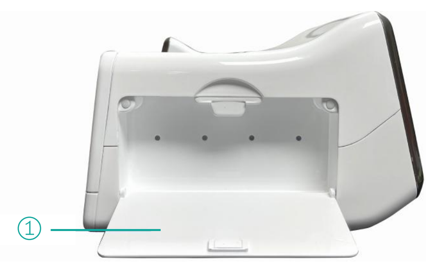

Figure 4
① The door of the disinfection box was opened

4. The right side of Lung Clear Device ZD-HY is equipped with a gas-liquid separator and an audio output port. The gas-liquid separator is used to store the liquid formed after condensation of discharged residual gas (see Figure 5);
5. The rear of the gas-liquid separator is powered on with a normally bright backlight source, and a water level line is arranged under the air inlet to facilitate observation of the water level. There is a catheter jack above the gas-liquid separator, which is used to connect the catheter, and an air outlet on the back is used to connect the main engine (see Figure 5);

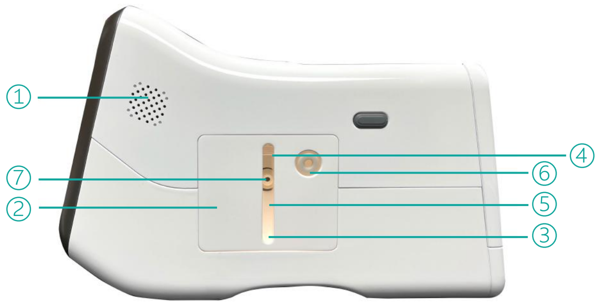

Figure 5

① Audio output
② Gas-liquid separator
③ Backlight source
④ Water level
⑤ Water level of observation window
⑥ Catheter jack
⑦ There are air outlets on the back

6. There is a gas-liquid separator button on the upper right of the catheter jack (see Figure 6);

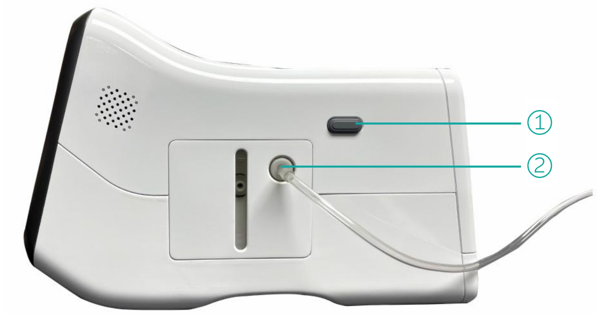

Figure 6
① Key switch of gas-liquid separator
② Catheter jack.

7. There is a pump body replacement opening at the bottom of the instrument, which is convenient for replacing the pump body. The pump body is fixed with four machine nails; The bottom of the pump body has a drain outlet, a noise-reducing sponge and a water-leaking cover, which are used to discharge the residual water gas of the pump body (see Figure 7, Figure 8 and Figure 9)

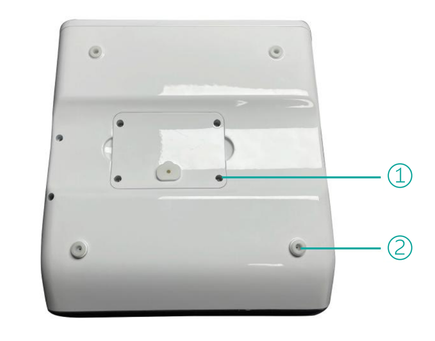

Figure 7

① Fix the pump body screw

② Floor mats

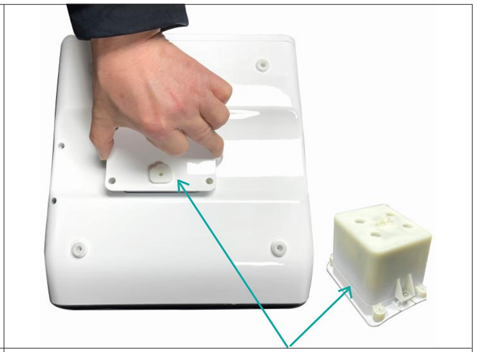

Figure 8

可更换泵体

Replaceable pump body

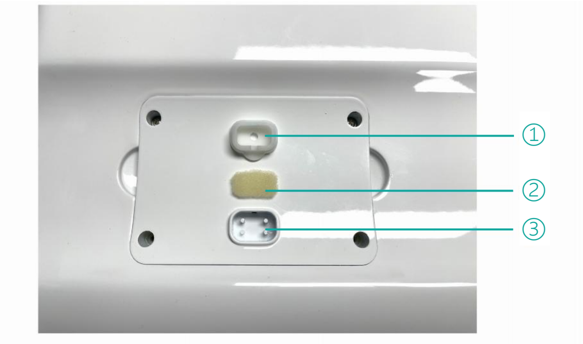

Figure 9

① Leakage cover

② Reducing mania sponge

③ Attensive cavity

8. Lung Clear Device ZD-HY is equipped with a self-packaged catheter with a length of 1.8 meters, a catheter plug at one end, a nasal plug at the other end and a fixed headgear in the middle (see Figure 15)

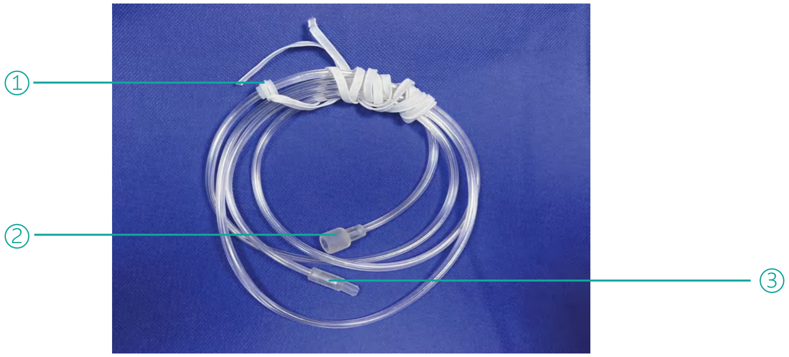

Figure 15

① Fixed headgear

② Nasal obstruction

③ Two-way plug

Product use:

1. Pre-use inspection: After you open the package, please check whether the instrument is damaged or missing accessories.

2. Open the catheter package, insert one end into the gas-liquid separator and one end into one nostril as shown in the figure, turn on the power supply, and place Lung Clear Device in a lower position (so as to facilitate the drainage of water in the catheter);

3. First meeting panel, Lung Clear Device panel is LCD screen, and the function buttons on the right side of the screen are 5 capacitive touch buttons (see Figure 1). After turning on the rear power switch, the front LCD screen lights up by default, the prompt tone prompts welcome words, and the backlight of the gas-liquid separator lights up;

4. The upper left is: timing, time and sub-icon; Below are the usage time accumulation icon and

5-bit numerical icon, followed by high, medium and low gear icons, regular mode, enhanced mode icons and disinfection icons; The right side of the isolator is a lung motion icon, which is a dynamic diagram. According to the selected gear, the mode changes accordingly, and the voice opening and closing icons are below;

5. Lung Clear Device starts to work in the strong gear state in the normal mode by default. Touch the start/pause button, and the instrument starts and pauses switching in the original working state;

6. When touching the gear key in the normal mode, switch between high, medium and low states (see Figure 10);

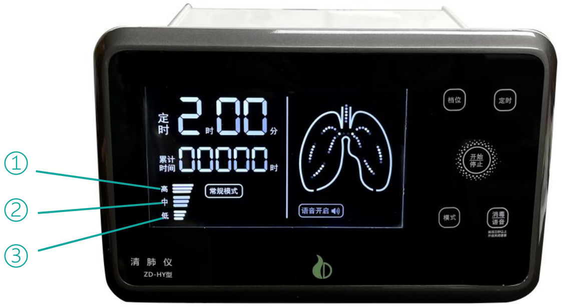

Figure 10

① High grade
② Mid-range
③ Resist

7. Touch the mode button to switch between normal mode and enhanced mode (see Figure 11);

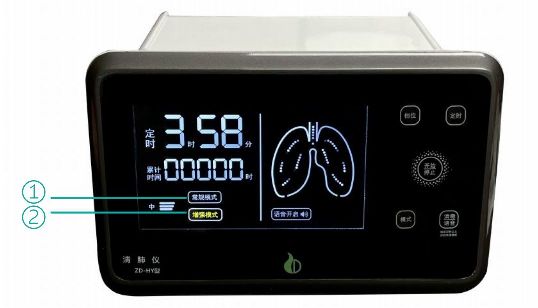

Figure 10

①Conventional mode
②Enhanced mode

8. In the enhanced mode, there is only one working state, no gear change and no gear icon display;

9. Select the timing. Touch the timing button and increase it by one hour every time it is pressed, which can accumulate the timing length for 4 hours. After three seconds, the screen displays a flashing state. When the timing length is reached, the machine stops working (but it has nothing to do with disinfection), and the timing icon no longer flashes; When the timing function is not selected, the timer icon for continuous working of the machine does not flash;

10. When the disinfection/voice button is touched, the ultraviolet disinfection lamp of the disinfection box starts to work, and the disinfection is stopped by touching again. Each disinfection lasts for ten minutes. After ten minutes, the disinfection sign on the panel stops flashing, and the disinfection is over. When the disinfection box is not closed, the disinfection lamp will not light up (see Figure 12);

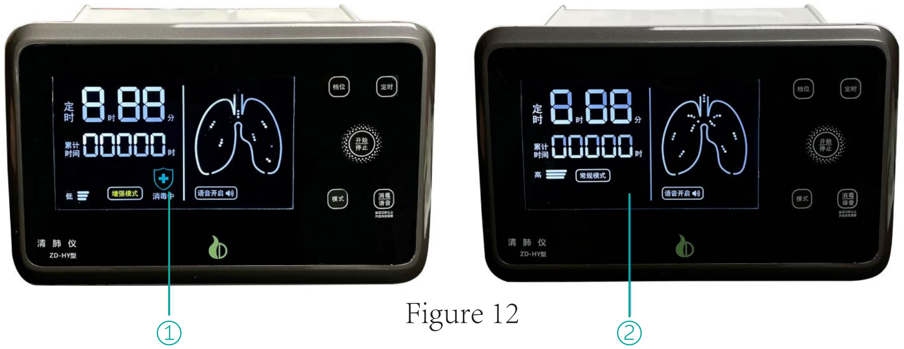

Figure 12

① In disinfection
② Not disinfected

11. Press the disinfection/voice button for 3 seconds to turn off or turn on

the voice. The startup defaults to the on state, and use the option to turn off the voice at night or during lunch break (see Figure 13);

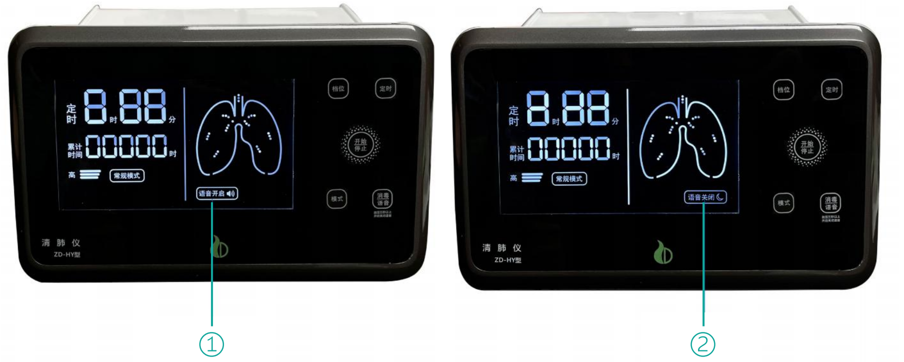

Figure 13

① Voice on
② Voice off

12. In use, there will be water flow into the gas-liquid separator through the conduit. The amount of water will be different according to factors such as gear height, use time, environmental temperature and individual differences. Generally, within two hours, the water level of the gas-liquid separator will not reach the water level. Observe the water level in the gas-liquid separator in time during use. When the water level exceeds the marked water level, it should be dumped in time to avoid flowing into the pump body, causing noise increase and shortening the ervice life of the pump body;

13. When you need to pause temporarily in use, you can touch the pause button. Repeated touch will start working. When you don't use it for a long time, you should turn off the back power switch button;

14. After turning off the power supply, take off the catheter, clean the gas-liquid separator in time (the flushing method will be described later), and pull away the power cord.

Installation and cleaning methods:

1. Lung Clear Device ZD-HY gas-liquid separator is installed by default, only need to cut the catheter package and take out the catheter; Insert the catheter plug into the catheter jack of the gas-liquid separator, and then gently insert the nasal plug at the other end of the catheter into one nostril; Finally, hang the headgear on the back head like wearing a mask (see Figure 16);

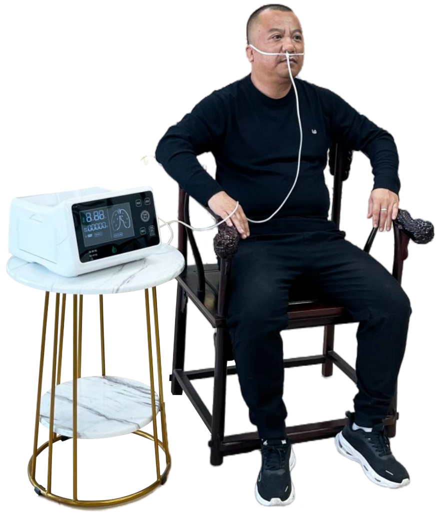

Figure 16

2. After use, please take off the headgear and nasal plug, press the switch of the gas-liquid separator, and the gas-liquid separator will tilt and pop up by itself. Lift the catheter up and take it off together with the gas-liquid separator for flushing (see Figure 14);

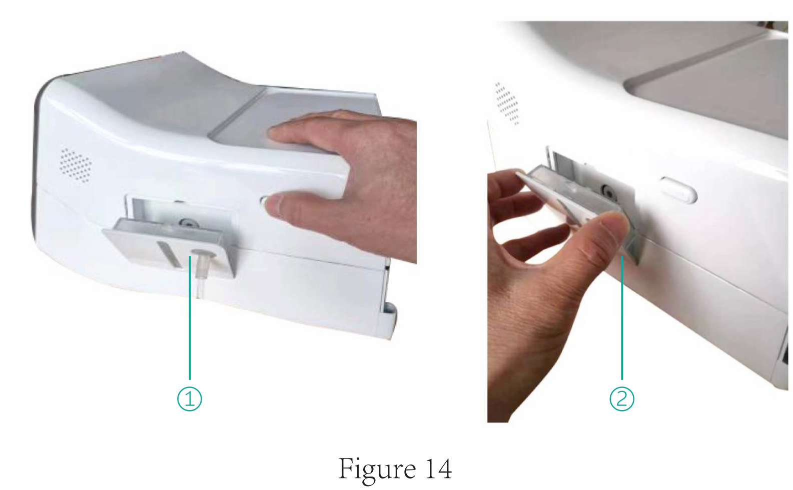

Figure 14

① The gas-liquid separator is opened
② Installation of gas-liquid separator

3. First, pull the plug on the catheter from the catheter jack of the gas-liquid separator, with the catheter jack facing down and the air outlet facing up, pour out condensed stored water, then pour water into the gas-liquid separator, shake and wash it, and then dry the water in the gas-liquid separator (see structural diagram);
4. Then align the nasal end of the catheter with the faucet and irrigate it inward. Lower the other end of the catheter to drain water. After washing, dry the water in the catheter. It is best to hang or put it in a disinfection box for disinfection;
5. Push the gas-liquid separator inward according to the clamping groove, and install it when the switch of the gas-liquid separator pops up and the gas-liquid separator is flat and firm (see Figure 14).

IX. Treatment time and course

Treatment time:

1) One of the principles of Lung Clear Device ZD-HY treatment is that the sputum excretion speed must be faster than the sputum secretion speed, and the sputum can be prevented from staying in the respiratory tract for a long time, which can dredge the respiratory tract and reduce the number of bacteria and viruses in the respiratory tract, thus effectively treating and controlling the incidence of infection;
2) When starting treatment, it must be used more often, and the sputum and residual gas blocked in the deep lung should be discharged. Use the "strong file" for 1-2 hours each time, more than 3-4 times a day, and use it for 6-8 hours a day; Those who can't tolerate it can choose "mid-range" to gradually apply it;
3) After the deep lung of the patient is cleaned up, when the respiratory function and asthma symptoms gradually improve, it can be used appropriately to prevent health care and promote the gradual recovery of respiratory function;
4) For patients with emphysema and asthma or hypoxia at night, it is best to use "mid-range" or "weak range" with the machine all night when sleeping at night, which can improve the hypoxia of patients at night, facilitate expectoration in the morning and speed up the recovery of respiratory function;
5) For patients with sticky sputum and difficulty in coughing, the use time should be controlled within 1 hour each time, and it should be used several times a day;
6) According to your own condition and the attenuation degree of lung function, you can take symptom improvement and self-comfort as the treatment scale;
7) Preventive health care should adhere to regular lung-clearing health care for 1-2 hours every day to reduce the attachment and deposition of harmful substances in the lungs and prevent the occurrence of occupa-

tional diseases and respiratory diseases. Course of treatment:
30 days is a course of treatment. It is recommended that mild patients choose 1-3 courses of treatment, while moderate and severe patients should insist on treatment for more than one year. Please use the specific course of treatment under the guidance of experts and doctors. Correct use and persistent treatment are the key to functional rehabilitation treatment. If you still have unclear specific problems, you can call the national unified consultation telephone number: 010-62563856 at any time.

X. Precautions

1. Critically ill patients should use it under the guidance of experts and doctors; It cannot be used at the same time as oxygen inhalation and nasal feeding patients;
2. Please install, use and clean in strict accordance with the methods introduced in the instructions after each use, and do not disassemble and assemble other parts of the instrument without permission to ensure your personal safety and curative effect;
3. Lung Clear Device ZD-HY helps to expel sputum. Sputum is coughed easily and autonomously through the mouth, not sucked by catheter! Please pay attention to observe the color, quantity, shape and viscosity of sputum before and after treatment. Transparent, thin and small
amount of normal sputum;
4. Lung Clear Device ZD-HY can be used by many people respectively, but the catheter should be used by special person, and tuberculosis patients should use one instrument alone;
5. The sealing ring on the gas-liquid separator should be checked frequently to prevent damage or fall off and leak air;
6. When disinfecting the catheter, try to control the water in the catheter dry and put it in the disinfection box for disinfection;
7. Lung Clear Device ZD-HY should be stored in a dry and ventilated room with an
ambient temperature of 15 ℃ to 30 ℃ to prevent severe impact, vibration and humidity;
8. Lung Clear Device ZD-HY power cord is designed for convenient storage, which is
beneficial to service life when pulling and returning lightly;
9. Please read the instructions carefully before use to understand the product operation, or the
users are medical staff with professional knowledge and trained and qualified personnel.

XI. Warm Tips and Maintenance Methods

1. Adjust the gear and mode according to the treatment needs, and the treatment can be started;
2. Please drink plenty of water before and after treatment, especially the sputum is sticky. Drinking water helps dilute the sputum, expel sputum smoothly, and avoid dry mouth and dry throat;
3. Please shut up and breathe as much as possible during use, and the two nostrils can be used interchangeably; Sitting, lying and leaning can be used;
4. When using Lung Clear Device ZD-HY, the flow rate per unit time in conventional mode and enhanced mode has little change, but the vibration amplitude of Lung Depurating Apparatus will increase. When sputum is difficult to discharge, the enhanced mode is more conducive to sputum excretion, and the strong gear in conventional mode is better for those with severe air blockage;
5. The gas-liquid separator can generally accommodate water stored in strong gear for about 2to 3 weeeks, but the water stored is determined by three factors: suction, indoor temperature and humidity in lungs. There-

fore, as long as the suction is normal, the amount of water generated during use will vary from person to person. Please dump the water stored in the separator in time to avoid the water level exceeding the water level at the lower end of the air inlet of the gas-liquid separator;

6. After use, be sure to pour out the liquid in the gas-liquid separator in time and clean it to avoid water inflow into the body and damage the instrument;

7. If mucus flows into the catheter during use, stop using it as soon as possible. Please don't worry, this is the result of negative pressure drainage in nasal cavity; At this time, you should immediately clean the mucus and flush the catheter to avoid mucus entering the gas-liquid separator, and also try to avoid mucus entering the catheter;

8. Lung Clear Device ZD-HY negative pressure pulsation therapy has no implantation for human body, no cross infection and good safety. The catheter can be flushed for many times. It is recommended to replace it once every 2-3 weeks, and the gas-liquid separator can be used for a long time;

9. The disinfection box can be used for users to disinfect the catheter. It is best to wash it with clear water and then put it in the disinfection box for disinfection. Hot water and alcohol cannot be used to avoid hardening;

10. Cough and expectoration during or after use are normal phenomena to promote expectoration. Please don't worry;

11. Garbage produced during the operation of equipment or equipment use shall be disposed of according to relevant local laws and regulations, and shall not be discarded at will to avoid cross infection.

XII. General Troubleshooting

1. The "purr" sound of the catheter indicates that the catheter is bent and blocked, which makes the drainage unsmooth. The catheter position should be straightened so that the condensed water can be smoothly discharged into the gas-liquid separator;

2. Regularly check whether the flow rate is normal. When the connected catheter is turned on, gently press the cheek with the nasal plug to feel whether there is strong suction or vibration, or insert the catheter into a small bottle cap filled with water to observe whether the suction is fast and powerful;

3. If the patient finds that the suction force of the machine is reduced, he can check whether there is a crack in the gas-liquid separator, whether the sealing rings of the catheter jack and air outlet fall off, whether the interfaces are inserted tightly, and whether the catheter is killed and bent. If there are no above problems, he should send the whole machine back to the company for maintenance.

4. If there is a crack in the gas-liquid separator, please contact the manufacturer for replacement in time;

5. If the catheter is found to be black and moldy after use, the catheter must be replaced in time;

XIII. Safety Warning

Check whether the power cord is damaged or exposed before use; Whether the plug has damp foreign bodies; Whether the socket connection is stable or not, ensure that it is used safely. Please do not disassemble the product with electricity without permission when it fails, which may cause electric shock risk.

XIV. Service life and maintenance of products

1. The normal service life of Lung Clear Device ZD-HY is 10 years (tentative, excluding wearing parts); In use, Lung Clear Device ZD-HY should not only wash the catheter and gas-liquid separator regularly, but also pay attention to: wash the gas-liquid separator carefully when not in use for a long time, dry it and then stick it on the main engine and place it in a ventilated and clean environment; It is recommended to replace the catheter (oxygen inhalation tube) for three months to prevent infection caused by

mildew and deterioration;

2. The pump body of Lung Clear Device ZD-HY is set as a wearing part. When the suction force of the self-inspection product becomes small (please consult the self-inspection method of suction force after-sales inspection), you can purchase and replace it by yourself: turn over Lung Clear Device, see that there are four screws fixed at the bottom center, unscrew the four fixing screws and pull them down, and then push in a new pump screw for fixing.

XV. Service Pledge

This product is guaranteed for one year from the date of sale, and materials will be charged for repair after one year, except for man-made damage (see Lung Clear Device Warranty Card for details).

Maintenance address: Room B 509, Building 1, No.2 Jinfu Road, Daxing District Economic Development Zone, Beijing

XVI. Transportation and storage conditions

Lung Clear Device ZD-HY should use closed transport vehicles during transportation to prevent severe impact; It should be stored in a dry and ventilated environment with an ambient temperature of -20 ~ 55℃ and humidity ≤ 93% to prevent violent vibration and humidity.

XVII. Manufacturer's Declaration on Electromagnetic Compatibility Notes:

1. The purchaser or user of Lung Clear Device ZD-HY shall use the equipment in theelectromagnetic environment specified in Table 201, 202, 204 and 206, otherwise the equipmentmay not work properly.

2. Portable and mobile RF communication equipment may affect the normal use of the equipment. Please use the equipment in the recommended electromagnetic environment.

Warning:

1. In addition to the accessories and cables provided by the manufacturer of Lung Clear Device ZD-HY (see attached table), the use of accessories and cables other than those specified may result in an increase in emission or a decrease in immunity of the equipment.

2. Lung Clear Device ZD-HY should not be used in close proximity or stacked with other equipment. If it must be used in close proximity or stacked, it should be observed and verified that it can operate normally under its used configuration.

Basic performance: Accuracy of suction flow and negative pressure range.

Table 201

Guideline and Manufacturer's Statement-Electromagnetic Emission		
Lung Clear Device ZD-HY is intended to be used in the following specified electromagnetic environments, and the purchaser or user shall guarantee its use in such electro-magnetic environments:		
Launch test	Conformity	Electromagnetic Environment-Guideline
Radio frequency emission GB4824	Group 1	The device uses RF energy only for its internal functions. Therefore, its radio frequency emission is very low, and the possibility of interference to nearby electronic equipment is very small.

Radio frequency emission GB4824	Category B	The device is suitable for use in all facilities, including domestic facilities and public low-voltage power supply networks directly connected to domestic residences.
Harmonic emission GB17625.1	Category A	
Voltage fluctuation/flicker emission GB17625.2	Conformity	

Table 202

Guidelines and Manufacturer's Statements-Electromagnetic Immunity			
The equipment is intended to be used in the following specified electromagnetic environment, and the purchaser or user shall guarantee its use in this electromagnetic environment:			
Immunity test	IEC60601 Test level	Coincidence level	Electromagnetic Environment-Guideline
Electrostatic Discharge (ESD) GB/T17626.2	± 6kV contact discharge ± 8kV air discharge	± 6kV contact discharge ± 8kV air discharge	The floor should be made of wood, concrete or ceramic tiles, and if the floor is covered with synthetic materials, the relative humidity should be at least 30%.
Electrical Fast Transient GB/T17626.4	±2kV for power cord ±1kV for input/output lines	±2kV for power cord ±1kV not applicable to input/output lines	The grid power supply should be of typical quality used in commercial or hospital environment
Surge GB/T17626.5	± 1kV line-to-line ± 2kV line to ground	±2kV for power cord ±1kV not applicable to input/output lines	The grid power supply should be of typical quality used in commercial or hospital environment
Voltage sag, short-term interruption and voltage change on power supply input line GB/T17626.11	< 5% UT, lasting 0.5 weeks (> 95% sag on UT) 40% UT, lasting 5 weeks (60% sag on UT) 70% UT, lasting 25 weeks (30% sag on UT) < 5% UT for 5s (> 95% sag on UT)	< 5% UT, lasting 0.5 weeks (> 95% sag on UT) 40% UT, lasting 5 weeks (60% sag on UT) 70% UT, lasting 25 weeks (30% sag on UT) < 5% UT for 5s (> 95% sag on UT))	The grid power supply should be of typical quality used in commercial or hospital environment. If the user of the device needs continuous operation during the power interruption, it is recommended that the device be powered by an uninterruptible power supply or battery.
Power frequency magnetic field (50/60 Hz) GB/T17626.8	3A/m	3A/m	The power frequency magnetic field should have the horizontal characteristics of the power frequency magnetic field in a typical commercial or hospital environment.
Note: UT refers to the AC network voltage before the test voltage is applied			

Table 204

Guidelines and Manufacturer's Statements-Electromagnetic Immunity

The equipment is intended to be used in the following specified electromagnetic environment, and the purchaser or user shall guarantee its use in this electromagnetic environment:			
Immunity test	IEC60601 Test level	Coincidence level	Electromagnetic Environment-Guideline
Portable and mobile RF communication equipment shall not be used closer than the recommended isolation distance to any part of the equipment, including cables. The distance shall be calculated by the formula corresponding to the transmitter frequency. Recommended isolation distance.			
Radio frequency conduction GB/T1762 6.6	3V (RMS) 150kHz~80MHz	3V (RMS)	$d= 1.2\sqrt{P}$
Radio frequency radiation GB/T1762 6.3	3V/m 80MHz～2.5GHz	3V/m	$d= 1.2\sqrt{P}$ 80MHz~800MHz<br>$d= 2.3\sqrt{P}$ 800MHz~2.5MHz<br>Where: P-based on the transmitter manufacturer's maximum rated output power, in watts (W); d-Recommended isolation distance in meters (m). The field strength of a fixed RF transmitter is determined by surveying the electromagnetic field and should be lower than the coincidence level in each frequency range. Interference may occur near devices marked with the following symbols.<br>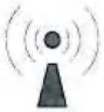

Note 1: At 80MHz and 800MHz frequency points, the formula of higher frequency band is adopted.<br>Note 2: These guidelines may not be appropriate for all cases, where electromagnetic propagation is affected by absorption and reflection from buildings, objects and the human body.
a) The field strength of stationary transmitters, such as base stations of wireless (cellular/cordless) telephones and terrestrial mobile radios, amateur radios, AM and FM radio broadcasts and television broadcasts, cannot theoretically be accurately predicted. For the purpose of evaluating the electromagnetic environment of stationary RF transmitters, a survey of the electromagnetic field should be considered. If the field strength of the place in which the equipment is located is measured to be above the applicable RF compliance level mentioned above, the equipment should be observed to verify its normal operation. If abnormal performance is observed, additional measures may be necessary, such as reorientation or position of the equipment.<br>b) The field strength should be less than 3V/m over the entire frequency range from 150kHz to 80MHz.

Table 206

Recommended isolation distance between portable and mobile radio frequency communication equipment and the equipment
The equipment is expected to be used in an electromagnetic environment where RF radiation disturbance is controlled. Depending on the maximum rated output power of the communication equipment, the purchaser or user can prevent electromagnetic interference by maintaining the minimum distance between the portable and mobile radio frequency communication equipment (transmitter) and the equipment as recommended below

Maximum rated output power W of transmitter	Isolation distance corresponding to different frequencies of transmitter/m		
	1.5kHz-80MHz $d=1.2\sqrt{P}$	80MHz-800MHz $d=1.2\sqrt{P}$	800MHz~2.5GHz $d=2.3\sqrt{P}$
0.01	0.12	0.12	0.32
0.1	0.38	0.38	0.38
1	1.2	1.2	2.3
10	3.8	3.8	7.3
100	12	12	23
For the transmitter maximum rated output power not listed in the above table, the recommended isolation distance d, in meters (m), can be determined by the formula in the corresponding transmitter frequency column, where Q is the transmitter maximum rated output power provided by the transmitter manufacturer, in watts (W).			
Note 1: At 80MHz and 800MHz frequency points, the formula of higher frequency range is adopted.<br>Note 2: These guidelines may not be appropriate for all cases, where electromagnetic propagation is affected by absorption and reflection from buildings, objects and the human body.			

XVIII. Description of Replaceable Parts

If you need to replace parts, please contact the manufacturer

XIX. Symbol Description

No.	Figure	Description
1	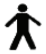	Type B application part
2	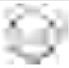	Disconnect (total power supply)
3		Turn on (total power supply)
4	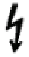	Dangerous voltage
5	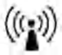	Non-ionizing radiation
6	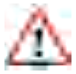	**Warning**<br>Indicates that if the instructions are not strictly followed, it may cause danger to patients or the system.
7	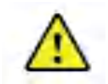	**Be careful**<br>Indicates that if the instructions are not strictly followed, it may cause danger to the system.
8	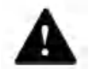	**Attention**<br>Indicates the situations that need to be paid attention to to ensure the optimal operation of the system.
9	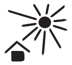	**Sunscreen**<br>Transport packages cannot be directly exposed to the sun
10	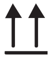	**Up**<br>The correct position of transport package is vertical upward
11	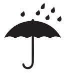	**Rainproof**<br>Packages are afraid of rain

XVIII. Description of Replaceable Parts

If you need to replace parts, please contact the manufacturer

XIX. Symbol Description

No.	Name	Specification	Quantity	Materials	Remarks
I	Lung Clear Device Host		1 台		
II	Random fittings				
1	Gas-liquid separator		1		Installed
2	Catheter (oxygen tube)	Single nose	1 set	Medical PVC	
3	Power cord	National standard 8-character power cord	1	Rubber-clad copper	
III	Random attachment				
1	Outer sealing ring of air nozzle		2	Medical silica gel	
2	Inner sealing ring of airnozzle		1	Medical silica gel	
3	Noise reducing cotton		1	Sponge	
IV	Random file				
1	Instruction for use of Lung Clear Device ZD-HY		1 piece		
2	Product certificate		1 piece		
3	Warranty card		1 piece		

Care for lung health and let lung breathe freely!

Date of preparation: January 2022 Version: V1.0

Revision date: July 2022 Version: V1.2
